# Social cognitive heterogeneity in schizophrenia: A cluster analysis

**DOI:** 10.1016/j.scog.2022.100264

**Published:** 2022-07-07

**Authors:** Anja Vaskinn, Kjetil Sundet, Beathe Haatveit

**Affiliations:** a Centre for Research and Education in Forensic Psychiatry, Oslo University Hospital, Norway; b Norwegian Centre for Mental Disorders Research, Institute of Clinical Medicine, University of Oslo, Norway; c Department of Psychology, University of Oslo, Norway; d Norwegian Centre for Mental Disorders Research, Oslo University Hospital, Norway

**Keywords:** Emotion processing, Heterogeneity, Theory of mind, Social perception

## Abstract

This study examined social cognitive heterogeneity in Norwegian sample of individuals with schizophrenia (n = 82). They were assessed with three social cognitive tests: Emotion in Biological Motion (emotion processing), Relationships Across Domains (social perception), and Movie for the Assessment of Social Cognition (theory of mind). Hierarchical and *k*-means cluster analyses using standardized scores on these three tests provided two clusters. The first cluster (68 %) had mild social cognitive impairments (<0.5 standard deviations below healthy comparison participants). The second cluster (32 %) had severe social cognitive impairments (>2 standard deviations below healthy comparison participants). Validity of the two social cognitive subgroups was indicated by significant differences in functioning, symptom load and nonsocial cognition. Our study shows that social cognitive tests can be used for clinical and cognitive subtyping. This is of potential relevance for treatment.

## 1 Introduction

Although cognitive decrements are present in schizophrenia, regardless of intellectual level (Vaskinn et al., 2014), there is great cognitive heterogeneity. Studies using different cognitive tests and different classification approaches have often identified three cognitive subgroups (Carruthers et al., 2019, Carruthers et al., 2021). One relatively intact group with only subtle cognitive impairment, a second group with moderate reductions and a third group with widespread cognitive dysfunction. Of note, there are also reports of four subgroups (Lewandowski et al., 2018). Researchers have arrived at these groups using clinical (Weickert et al., 2000) or data-driven (Vaskinn et al., 2020) classification methods, often cluster analyses. Differences in brain structure (Weinberg et al., 2016), symptomatic load (Vaskinn et al., 2020), and functional impairment (Vaskinn et al., 2020) support the validity of these groups (Carruthers et al., 2021).

Most such classification studies have focused on premorbid and/or current nonsocial cognition, i.e., performance on standard neuropsychological tests. It is less clear if other cognitive measures can classify individuals with schizophrenia in a meaningful way. A few studies used performance on social cognitive tests to group participants using cluster analyses, distinguishing three groups. Rocca et al. (2016) identified three groups with social cognitive impairments compared to healthy controls, but of different magnitude. The “unimpaired” cluster (42 %) had subtle deficits (<0.5 SD), whereas the deficits of the “impaired” (50.4 %) and “very impaired” (7.5 %) clusters were substantial. Similarly, Etchepare et al.'s (2019) “high social cognition” cluster (47.9 %) only differed significantly from healthy controls on one of the social cognition measures, whereas their “medium” (28.7 %) and “low social cognition” (23.4 %) clusters evidenced mild-moderate or substantial social cognitive deficits. The three clusters did not show different social cognitive profile patterns, but rather differences in *level* of social cognitive performance. Hajduk et al. (2018) is the only study that has identified a cluster that appears to be truly intact. Their “intact” (26 %) cluster had in fact social cognitive performance above healthy controls, at least for some of the utilized tests. The two other clusters had “mild” (43 %) or “severe” social cognitive impairment (31 %).

We wanted to further explore whether social cognitive measures can identify heterogeneity and subjected a schizophrenia sample's performance on a battery of social cognitive tests to empirical classification. Given previous findings, we hypothesized that three groups would emerge, and that these groups would differ beyond their social cognitive test results.

## 2 Methods

### 2.1 Participants

Eighty-two participants with a SCID-verified DSM-IV diagnosis of schizophrenia (n = 65) or schizoaffective disorder (n = 17) from a previous study (Vaskinn et al., 2018) at the NORMENT center in Oslo, Norway were included. They were recruited from hospitals in Oslo and Akershus counties. Only persons with IQ ≥ 70 and without a history of head injury and/or neurological disorder were eligible for participation. All participants had Norwegian as their mother tongue or had received all compulsory schooling in Norway. A comparison sample of n = 124 healthy controls randomly selected from official population records was also included. After having received information about the study, participants signed the consent form. The regional ethics committee approved the study. See Table 2 for demographic information.

### 2.2 Measures

#### 2.2.1 Social cognitive tests

Three social cognitive tests were administered. The Emotion in Biological Motion (EmoBio) (Heberlein et al., 2004) test is a point-light display measure of the ability to perceive emotions in moving bodies. The Movie for the Assessment of Social Cognition (MASC) (Dziobek et al., 2006) assesses cognitive and affective theory of mind (ToM). Social perception was measured with the Relationships Across Domains (RAD) test (Sergi et al., 2009). We have performed Norwegian validations of all three tests (Fretland et al., 2015; Vaskinn et al., 2016; Vaskinn et al., 2017) and used their respective total score in our analyses. Scores were standardized based on the performance of healthy controls (EmoBio n = 65, MASC n = 71, RAD n = 56).

#### 2.2.2 Nonsocial cognitive tests

We measured IQ with the 2-subtest version of the Wechsler Abbreviated Scale of Intelligence (Wechsler, 2007). Other cognitive functions were assessed with Matrics Cognitive Consensus Battery (MCCB: Nuechterlein and Green, 2009). We used the total score as well as the scores of the nine nonsocial cognitive tests (see Table 2).

#### 2.2.3 Clinical measures

Positive and negative symptoms were assessed with the Positive and Negative Syndrome Scale (PANSS) (Kay et al., 1987).

#### 2.2.4 Functioning

We measured social functioning with the self-report Social Functioning Scale (SFS: Birchwood et al., 1994) and used role-play tests to index social (Assessment of Interpersonal Problem-Solving Skills, AIPSS: Donohoe et al., 1990) and nonsocial (UPSA-BN: Mausbach et al., 2007) functional capacity.

### 2.3 Statistical analyses

The cluster analyses were conducted in multiple steps using the standardized EmoBio, RAD and MASC test scores of the schizophrenia sample. We started by subjecting the scores to a hierarchical cluster analysis with complete linkage (furthest neighbor) and squared Euclidian distance, providing a dendrogram and a scree plot (of the agglomeration coefficients) for visual inspection. Next, K-mean cluster analyses were run, for two, three and four clusters. Based on cluster membership from these K-means cluster analyses, VRCs (variance ratio criterions: Calinski and Harabasz, 1974) were calculated for the two-, three-, and four-cluster solutions as an empirical test of the cluster solution. VRCs are considered among the best validation criteria for cluster analyses (Vendramin et al., 2010) and are calculated using the following formula:

$$\frac{{\mathrm{SS}}_{B}}{{\mathrm{SS}}_{W}}\times \frac{N-k}{k-1}$$

where N is number of individuals, *k* is number of clusters, SS_B_ is between-cluster variation and SS_W_ is within-cluster variation. Pooled between and within cluster sum of squares (SS_B_ and SS_W_) for the three social cognitive tests were used. A higher VRC is indicative of a better cluster solution (Calinski and Harabasz, 1974; Milligan and Cooper, 1985). The final cluster solution was based on the dendrogram, scree plot and the VRCs, combined. Thereafter, we attempted to validate the clusters by comparing them on measures of nonsocial cognition (MCCB) and functioning (SFS, AIPSS, UPSA-BN) using analyses of variance (ANOVAs). Last, we examined if social cognitive differences remained after controlling for nonsocial cognition (MCCB total), using a multivariate ANCOVA (MANCOVA).

## 3 Results

**Fig. 1:**
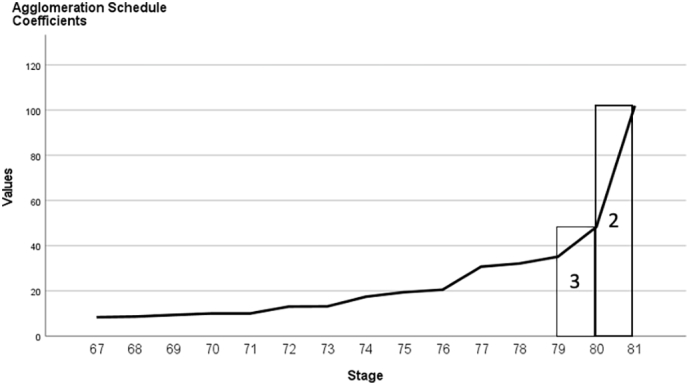
Scree plot of agglomeration coefficients.

**Fig. 2:**
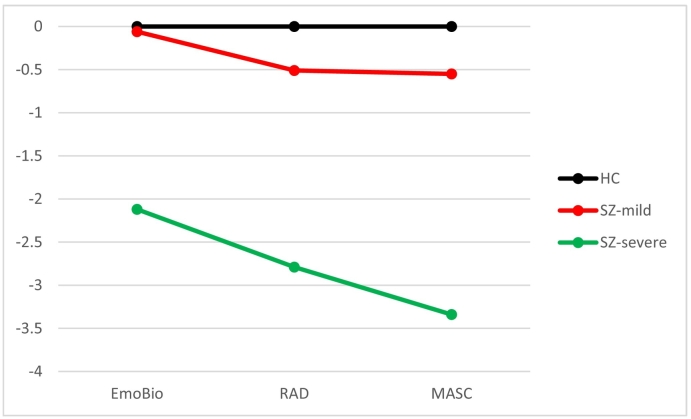
Social cognitive profile (z-scores).

The scree plot was ambiguous (see Fig. 1), but both the dendrogram and the VRCs indicated two clusters. The VRC for the two-cluster solution (73.85) was much higher than the VRCs for the three- and four-cluster solutions (47.94 and 46.95). The smallest cluster in the three-cluster solution consisted of 9 individuals (11.0 %). Two clusters, or social cognitive subgroups, were created. The first cluster had mild social cognitive impairments, scoring within approximately 0.5 standard deviations of healthy controls, and consisted of 56 individuals (68.3 %). The other cluster, with 26 individuals (31.7 %), had severe social cognitive impairments (>2 standard deviations below healthy participants). Their social cognitive profiles are presented in Fig. 2, based on z-scores in Table 1.

**Table 1:** Standardized scores (z) on the social cognitive tests in participants with schizophrenia.

	Total sample n = 82 M (SD)	Cluster 1 Mild impairment n = 56 M (SD)	Cluster 2 Severe impairment n = 26 M (SD)
EmoBio	−0.996 (1.77)	−0.06 (1.00)	−2.12 (1.32)
RAD	−1.235 (1.62)	−0.51 (1.04)	−2.79 (1.56)
MASC	−1.432 (1.76)	−0.55 (1.00)	−3.34 (1.51)

**Fig. 3:**
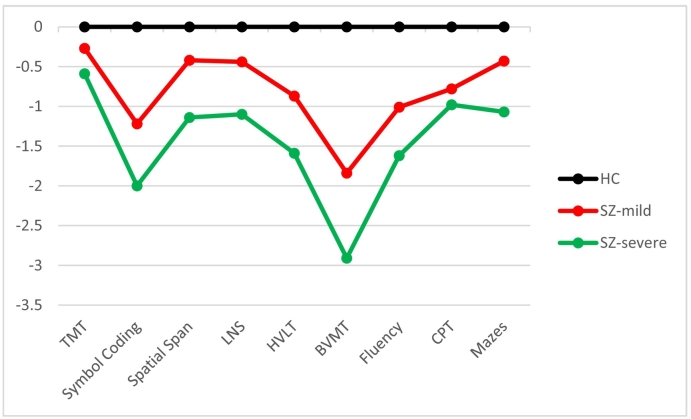
Nonsocial cognitive profile (z-scores).

The mild impairment cluster had higher IQ, longer education, and milder symptoms than the severe impairment cluster. Compared to healthy controls, the severe impairment cluster had impairments for all nonsocial cognitive tests. The mild impairment cluster did not differ significantly from healthy controls on Trail Making Test or NAB Mazes. Also, for these tests and CPT, they did not perform significantly different from the severe impairment cluster. For self-reported functioning (SFS) there was no difference between the two schizophrenia groups, but the severe impairment group had significantly worse performance on the functional capacity measures (AIPSS, UPSA-BN). See Table 2 for details and specific numbers; Fig. 3 for nonsocial cognitive profiles. Significant cluster differences for social cognition remained after controlling for nonsocial cognition (Wilk's Lambda = 0.314, F = 56.02, p < 0.001, η_p_^2^ = 0.69).

**Table 2:** Demographics, neuropsychological test performance, and functioning in healthy control participants and social cognitive subgroups with schizophrenia. Clinical symptoms in social cognitive subgroups with schizophrenia.

	Healthy control participants n = 124	Cluster 1: Mild impairment n = 56	Cluster 2: Severe impairment n = 26	Statistic	Post-hoc (Scheffe)	Effect size
Demographics						
Age	29.1 (7.6)	28.7 (8.9)	30.9 (8.0)	F = 0.71, ns	–	–
Sex (males/females)	76/48	35/21	19/7	x^2^ = 1.30, ns	–	–
Ecudation	14.3 (2.4)	12.5 (2.6)	11.4 (2.1)	F = 23.11, p < 0.001	HC > 1, 2	η_p_^2^ = 0.19
WASI IQ	111.5 (10.4)	104.1 (11.3)	91.4 (13.7)	F = 37.43, p < 0.001	HC > 1 > 2	η_p_^2^ = 0.27

Clinical symptoms						
PANSS positive	–	13.2 (4.1)	16.1 (5.4)	t = −2.75, p = 0.007	–	Cohen's *d* = 0.65
PANSS negative	–	13.8 (4.7)	16.9 (5.9)	t = −2.53, p = 0.013	–	Cohen's *d* = 0.60

Nonsocial cognition (T-scores)						
MCCB total score^1^	50.1 (6.1)	42.3 (7.0)	36.1 (5.9)	F = 67.93, p < 0.001	HC > 1 > 2	η_p_^2^ = 0.40
Trail Making Test	46.0 (11.9)	42.8 (10.1)	39.0 (9.4)	F = 4.95, p = 0.008	HC > 2	η_p_^2^ = 0.05
BACS Symbol Coding^2^	47.7 (10.6)	34.8 (9.5)	26.5 (7.0)	F = 67.23, p < 0.001	HC > 1 > 2	η_p_^2^ = 0.40
HVLT^2^	51.5 (9.8)	43.0 (8.5)	35.9 (8.0)	F = 38.84, p < 0.001	HC > 1 > 2	η_p_^2^ = 0.28
WMS Spatial Span	53.5 (10.3)	49.2 (10.4)	41.8 (10.0)	F = 14.82, p < 0.001	HC > 1 > 2	η_p_^2^ = 0.13
NAB Mazes	52.1 (9.0)	48.2 (11.7)	42.5 (13.0)	F = 10.32, p < 0.001	HC > 2	η_p_^2^ = 0.09
BVMT^2^	51.6 (9.0)	35.0 (12.2)	25.4 (7.8)	F = 106.94, p < 0.001	HC > 1 > 2	η_p_^2^ = 0.51
Semantic fluency	59.0 (10.5)	48.4 (10.9)	42.0 (10.6)	F = 37.57, p < 0.001	HC > 1 > 2	η_p_^2^ = 0.27
CPT^1^	45.5 (9.2)	38.3 (10.3)	36.5 (9.0)	F = 16.81, p < 0.001	HC > 1, 2	η_p_^2^ = 0.14
LNS	45.1 (9.1)	41.1 (9.6)	35.1 (8.0)	F = 14.34, p < 0.001	HC > 1 > 2	η_p_^2^ = 0.12

Functioning						
SFS^3^	122.7 (6.3)	104.1 (8.4)	106.8 (10.1)	F = 139.13, p < 0.001	HC > 1, 2	η_p_^2^ = 0.58
UPSA-BN^4^	–	77.2 (11.3)	69.1 (13.4)	t = 2.83, p = 0.006	–	Cohen's *d* = 0.68
AIPSS receiving	–	74.7 (18.1)	65.7 (20.7)	t = 2.01, p = 0.048	–	Cohen's *d* = 0.48
AIPSS processing	–	55.7 (20.6)	44.9 (24.6)	t = 2.08, p = 0.041	–	Cohen's *d* = 0.49
AIPSS sending	–	57.5 (19.7)	41.2 (14.5)	t = 3.76, p < 0.001	–	Cohen's *d* = 0.89

1 HC n = 123. ^2^ For this nonsocial cognitive test, the difference between cluster 1 and 2 in the post-hoc test remained significant after correcting for multiple comparisons: 0.05/9 subtests = 0.006 new p-level. ^3^ HC n = 120. ^4^ cluster 1: n = 55, cluster 2: n = 25. For UPSA-BN and AIPSS numbers indicate percentage correct.

## 4 Discussion

This cluster analytic study of social cognitive heterogeneity in schizophrenia provided two clusters or social cognitive subgroups. The validity of the subgroups was confirmed through significant differences in nonsocial cognitive performance, symptomatic load and in functioning. Thus, we found that social cognitive measures can identify meaningful subgroups. The number of identified subgroups contrasted with our hypothesis, drawn from the existing literature. We believe there might be a few possible explanations.

We may not have succeeded in recruiting participants from the relatively intact cluster that other studies identified. Such individuals are probably few - as cognitive decrements are so common in schizophrenia (Keefe et al., 2005) - and larger samples than ours may be needed for them to be classified as a separate cluster. The MCCB results support this explanation. For three of the MCCB tests our “mild impairment” cluster had deficits (*T* < 40), on one of which they did not differ significantly from the “severe impairment” cluster (CPT).

Another possibility is that our social cognitive battery is not particularly suitable when it comes to differentiating subgroups. In a previous study, we have speculated that our battery may not represent lower-level social cognition very well (Vaskinn et al., 2021). Perhaps the addition of a measure involving clear low-level processes would impact on the number of identified clusters.

We identified different levels of social cognitive impairment in schizophrenia of relevance for clinical subtyping and for treatment.

## Funding source

This work was supported by grants from the 10.13039/501100006095South-Eastern Norway Regional Health Authority (grants #2010007 and #2017069 to AV), the 10.13039/501100005416Research Council of Norway (grant #223273), and the Extra Foundation of Norway.

## CRediT authorship contribution statement

**Anja Vaskinn**: Conceptualization; Methodology; Data Acquisition; Resources; Formal analysis; Writing – Original Draft, Writing – Review and Editing, Funding Acquisition. **Kjetil Sundet**: Conceptualization; Methodology; Resources; Writing – Review and Editing, Funding Acquisition **Beathe Haatveit**: Methodology; Data Acquisition; Formal analysis; Writing – Original Draft, Writing – Review and Editing,

## Declaration of competing interest

Dr. Vaskinn has received consulting fees from VeraSci, Inc. The other authors report no conflict of interests.
